# The cyclin-like protein, SPY1, regulates the ERα and ERK1/2 pathways promoting tamoxifen resistance

**DOI:** 10.18632/oncotarget.15578

**Published:** 2017-02-21

**Authors:** Rosa-Maria Ferraiuolo, Janice Tubman, Indrajit Sinha, Caroline Hamm, Lisa Ann Porter

**Affiliations:** ^1^ Department of Biological Sciences, University of Windsor, Windsor, ON N9B 3P4, Canada; ^2^ Windsor Regional Hospital, Windsor, ON N8W 1L9, Canada; ^3^ Acenzia Inc, Tecumseh, ON N9A 6J3, Canada

**Keywords:** Cdk, cyclin, cell cycle, estrogen, tamoxifen

## Abstract

The Ras/Raf/MEK/ERK pathway conveys growth factor and mitogen signalling to control the phosphorylation of a plethora of substrates regulating proliferation, survival, and migration. The Ras signalling pathway is frequently associated with poor prognosis and drug resistance in various cancers including those of the blood, breast and prostate. Activation of the downstream effector ERK does not always occur via a linear cascade of events; complicating the targeting of this pathway therapeutically. This work describes a novel positive feedback loop where the cell cycle regulatory factor Spy1 (RINGO; gene *SPDYA*) activates ERK1/2 in a MEK-independent fashion. Spy1 was originally isolated for the ability to stimulate *Xenopus* oocyte maturation via a MAPK-signalling pathway and is known to override apoptosis triggered by the DNA damage response. We demonstrate that mammalian Spy1-mediated ERK activation increases ligand-independent phosphorylation and activation of estrogen receptor α, correlating with a decrease in tamoxifen sensitivity. This could define a novel druggable mechanism driving proliferation and resistance in select cancers.

## INTRODUCTION

Overall 5 year survival rates for breast cancer have increased by almost 20% since 1975, largely because of improved screening and drugs developed against estrogen signalling (ie. tamoxifen) and the Her2/Neu receptor (ie. trastuzumab) [1]. Despite these advances, a subset of patients either progress to, or initially present with, cancers that are unresponsive to current targeted therapies [2, 3]. As such, breast cancer remains the second leading cause of death from cancer among women [1]. Determining the mechanisms regulating the initiation and/or progression to a drug resistant status represents a current challenge in the breast cancer field.

This work focused on a cell cycle protein coined Spy1 (Speedy, RINGO) (gene *SPDYA*) that is elevated in a number of human cancers, including invasive carcinoma of the breast, as well as cancers of the liver, brain, and blood [4–12]. In the breast, Spy1 levels are elevated during proliferative stages of mammary development (puberty and pregnancy) and forced expression can drive mammary tumorigenesis [13]. Spy1 is one member of a family of ‘cyclin-like’ proteins that are expressed and degraded in a cyclic manner and are able to directly bind and activate the cyclin dependent kinases (CDK)s [14–16]. Spy1 functions in an atypical manner to classical cyclins in that it binds to both the G1/S and G2/M CDKs and directs phosphorylation of non-canonical CDK substrates [12, 17, 18]. Activation of the CDKs by Spy1 occurs independent of phosphorylation within the T-loop and dephosphorylation on defined inhibitory residues [17]. Further, Spy1 directly binds and promotes the degradation of the CDK inhibitor, p27^Kip1^ [8, 19]. Hence, Spy1 supports rapid progression through the cell cycle even in the face of senescence and apoptotic-inducing stimuli [4, 5]. This suggests a mechanism by which Spy1 overrides cell-cycle induced apoptosis caused by therapeutic agents, which could support drug resistance.

Proliferative programs in the breast, both during normal development and breast cancer, are in part dictated by signalling through the steroidal estrogen receptor alpha (ERα). ERα changes into an active conformation upon binding to the ligand estradiol (E2) [20]. Classical ER activation promotes receptor homodimerization, nuclear translocation and subsequent DNA binding to estrogen response elements (EREs) to regulate the expression of various genes [21]. ‘Non-classical’ genomic signalling also exists where the E2-ERα complex binds transcription factors to regulate genes lacking an ERE, like Cyclin D1 [22–25]. ER dimers, activated by E2 or other growth factors, can also interact and form complexes directly with G-proteins, receptor tyrosine kinases, and non-receptor tyrosine kinases [26]. Non-classical ER signalling can thereby trigger signal transduction pathways such as Ras/mitogen-activated protein kinase (MAPK) and phosphoinositide 3-kinase (PI3K/Akt) [27]. Collectively, ERα works via these diverse mechanisms to promote breast cell growth and survival [20, 28].

Tamoxifen functions by competitively binding to the ligand binding domain (LBD) of ERα, altering its conformation such that it can no longer bind to E2, hence, preventing E2 proliferative signalling [29]. The binding efficiency of tamoxifen can be altered by the phosphorylation status of residues within ERα capable of inducing ligand independent signalling [30, 31]. Phosphorylation on serine (S) 118 by extracellular signal-regulated kinases (ERK)1/2 is one prominent example of such a modification. S118 phosphorylation promotes hypersensitivity to E2 and decreases ERα affinity for tamoxifen [30, 32–35]. ERK1/2 is the final kinase at the end of the Ras/MAPK signalling cascade, succeeding Ras, Raf and MEK activation. Given that non-classical ERα signalling can activate a MAPK cascade, this represents a feedback mechanism enforcing activity of the ER [23, 24, 36–40].

The Ras-Raf-Mek-ERK cascade is hyperactivated in approximately 30% of human cancers; a large percentage characterised by a mutation in either the Ras or Raf genes [41, 42]. Constitutively activated MEK1/2 is frequently seen in cancer cell lines, contributing to increased cell survival, migration and transformation [42]. Overexpression and hyperphosphorylation of ERK1/2 has been seen in various cancers, including hepatocellular carcinoma and breast cancer [41, 42]. Pharmacological intervention upstream of ERK1/2 has received considerable focus; however, to date clinical results are largely underwhelming, with preclinical and clinical documentation showing a development of acquired resistance shortly after receiving treatment [43, 44]. Resistance is largely associated with re-activation of ERK1/2 signalling [45]. As such, specific inhibitors of ERK1/2 have become a focus over the last 5 years. Use of an ERK1/2 inhibitor can overcome acquired resistance to both BRAF and MEK1/2 inhibitors in melanoma, breast and colon cancer cell lines [45, 46]. This exciting data has led to the introduction of the ERK1/2 inhibitor into phase I clinical trials for solid tumours [45, 47, 48]. Understanding the activation of all components of this pathway influences the successful intervention of a large number of cancers, including breast cancer.

## RESULTS

### Spy1 is upregulated downstream of the estrogen receptor

**Figure 1:**
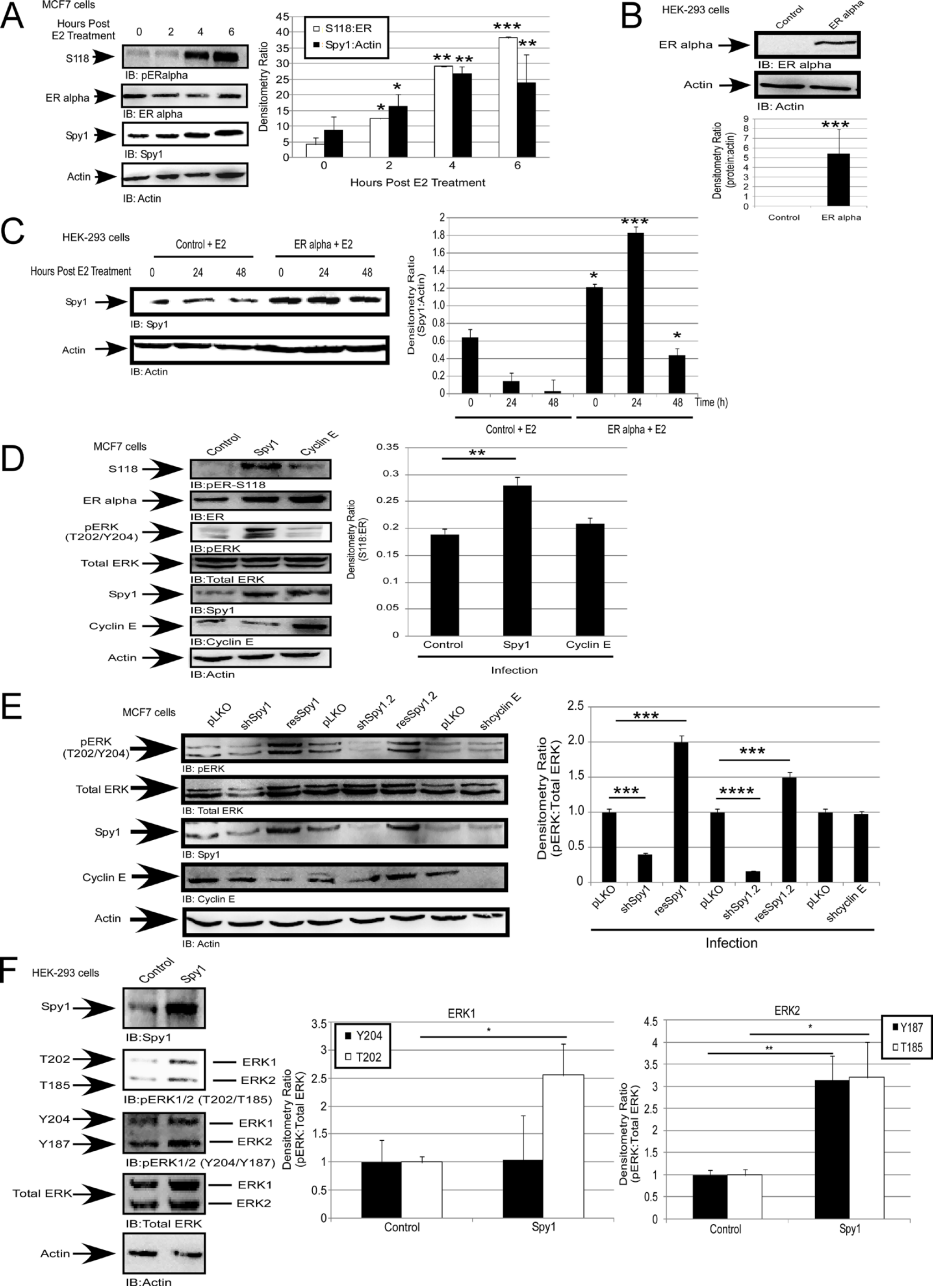
Spy1 is upregulated downstream of the estrogen receptor (**A**) MCF7 cells were treated with 50 nM of estradiol (E2) or vehicle control (DMSO) over the indicated time course. Representative blot (left), densitometry averages Spy1 and S118 (right). (**B**–**C**) Hek-293 cells were transfected with pEGFP-C1-ERα. B-Representative blot confirming expression. C-Treatment with 50 nM of E2 over the indicated time course. Representative blot (left), densitometry averages for Spy1 (right). (**D**) MCF7 cells infected with control, Spy1, or Cyclin E1.(**E**) Cells were infected pLKO, 2 constructs of shSpy1.1, shSpy1.2, shCyclin E, or rescue vectors, followed by SDS-PAGE and IB. Representative blot (left), densitometry averages for relative ER-S118 (right). (**F**) Representative blot (upper panel) showing phosphorylation status of ERK1 and ERK2 using phosphor-specific antibodies for pERK threonine or tyrosine sites. Lower panel depicts quantification of both sites for either ERK1 (left) or ERK2 (right). Error bars reflect SE between at least 3 separate experiments. Student's *t*-test was performed; *p < 0.05, **p < 0.01, ***p < 0.001.

Spy1 is upregulated downstream of c-Myc and developmental expression patterns mimic that seen with c-Myc [13, 49]. Given that c-Myc is upregulated downstream of ERα [50]; we used ERα-positive MCF7 breast cancer cells to determine whether Spy1 protein is also regulated downstream of ERα. Spy1 protein levels were elevated over a period of 1–6 hours following E2 treatment (Figure 1A). Protein levels were also elevated in response to E2 following reconstitution of ERα in ER negative HEK-293 cells (Figure 1B and 1C). These data indicate that upregulation of Spy1 protein levels occurs downstream of the estrogen signalling pathway. Overexpression of Spy1 in ER-positive MCF7 cells demonstrates a significant increase in the phosphorylation of the ER at S118 (Figure 1D). To determine if this was due to enhanced proliferation through G1/S, the G1/S cyclin, Cyclin E1, was overexpressed and phosphorylation of ER-S118 measured (Figure 1D). Elevated levels of Cyclin E1 had no significant change in ER-S118 phosphorylation.

Spy1 is capable of promoting the activation of the MAPK pathway when injected into unfertilized *Xenopus* oocytes [16], and S118 is phosphorylated by several kinases including ERK1/2 of the MAPK pathway [30, 33, 51]. We measured the activity of ERK (phospho-T202/Y204; pERK) in the presence of overexpressed Spy1 and found a significant increase in the level of phospho-ERK (Figure 1D), this was also seen in other cell systems (Supplementary Figure 1A–1B). While a slight increase in phosphorylation was also seen with Cyclin E1 overexpression, this difference was not statistically significant. These data support that activation of ERK, seen downstream of Spy1 overexpression, is not a generalized effect due to cell proliferation. To determine if Spy1 is a necessary mediator of ERK activation, HEK-293 cells were infected with shRNA lentivirus targeting two separate regions of the Spy1 mRNA (shSpy1.1, shSpy1.2). shRNA against Cyclin E1 was also used to address the essentiality of classical cyclin-CDK activation (shCyclinE) and a pLKO-shScrambled control (pLKO). Both of the shSpy1 constructs significantly decreased endogenous activated ERK levels (Figure 1E and Supplementary Figure 1C); this effect was not noted with shCyclinE treatment despite successful knockdown (Figure 1E and Supplementary Figure 1C; left panel representative blot). Spy1 effects were reversed by a rescue construct, showing specificity of the sh-targeting (resSpy1; Figure 1E). These results support that Spy1 is a required component for activation of ERK1/2 in this cell culture system.

To determine whether one of ERK1 or ERK2 was preferentially affected by Spy1, bands were separated to easily differentiate the family members and blotted with phospho-threonine or phospho-tyrosine specific antibodies to recognize ERK1-T202/ERK2-T185 and ERK1-Y204/ERK2-Y187 (Figure 1F). Our results show that Spy1 significantly increases the level of phosphorylation on both ERK1 and ERK2 with statistically consistent results for the threonine site in each protein. For the remainder of the experiments we focused on the average phosphorylation status of these proteins using the general T/Y-ERK phospho-specific antibody.

### Spy1 activation of ERK1/2 is dependent on CDK activation

**Figure 2:**
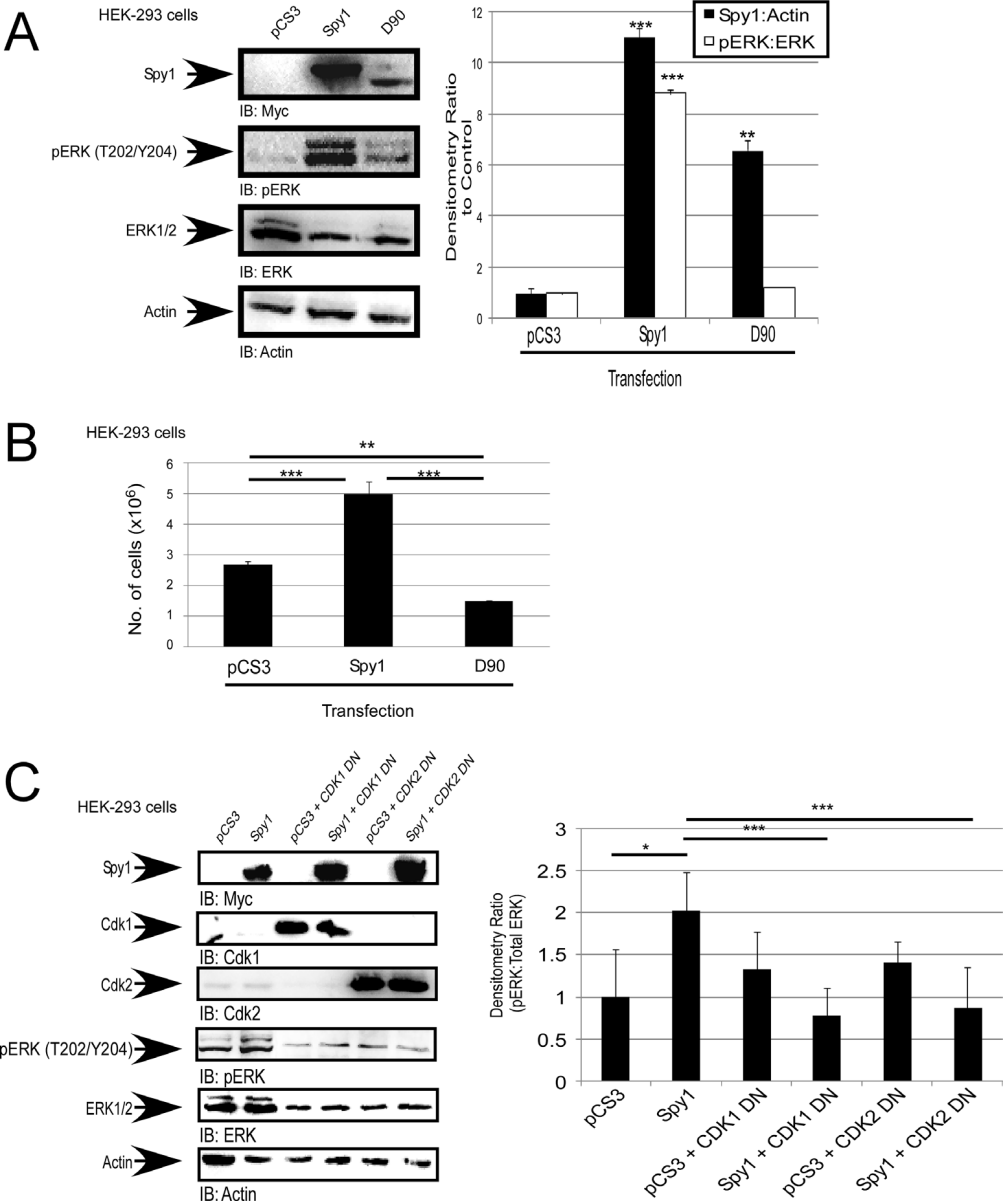
Spy1-mediated ERK phosphorylation is CDK dependent (**A**–**C**) Hek-293 cells were transfected with the indicated constructs (along top of each representative blot and X-axis of each graph, including the empty vector control (pCS3). (A) Representative blot (left). Densitometry for Spy1 or pERK over multiple experiments (right). (B) Trypan blue exclusion assay was performed after 24 hours of incubation, total cell numbers presented. (C) Representative blot (left). Densitometry (right) as represented on Y-axis. Error bars reflect SE between at least 3 experiments. Student's *t*-test was performed; *p < 0.05, **p < 0.002, ***p < 0.001.

Using a previously characterized Spy1-CDK non-binding mutant (Spy1-D90A) [17], we questioned whether the direct binding between Spy1 and the CDK is essential for activation of ERK1/2. Transient transfection with wild-type Spy1 shows a significant increase in the activation of ERK1/2 (Figure 2A), and a significant increase in proliferation, as compared to control and D90 transfected cells (Figure 2B). These data support the hypothesis that the activation of ERK1/2 is dependent on Spy1-mediated CDK activity. It is notable that altered migration of the Spy1-D90A mutant on SDS page gel has been consistently reported in the literature [17]. Spy1 can bind to both CDK1 and CDK2 [6, 12, 17]. To determine which CDK is most influential on Spy1-activated ERK, cells were transfected with Myc-tagged Spy1 and low levels of either an HA-tagged CDK1or CDK2 dominant negative (DN) vector (CDK1 DN or CDK2 DN), or relevant controls. The concentration of DN vector transfected did not significantly impair growth alone; however, both CDK1 and CDK2 DN vectors significantly impaired the ability of Spy1 to activate ERK1/2 (Figure 2C). Collectively, this data supports that Spy1-mediated phosphorylation of ERK requires at least one of the CDKs to be present and bound.

### Spy1-mediated ERK1/2 activation is MEK-independent

**Figure 3:**
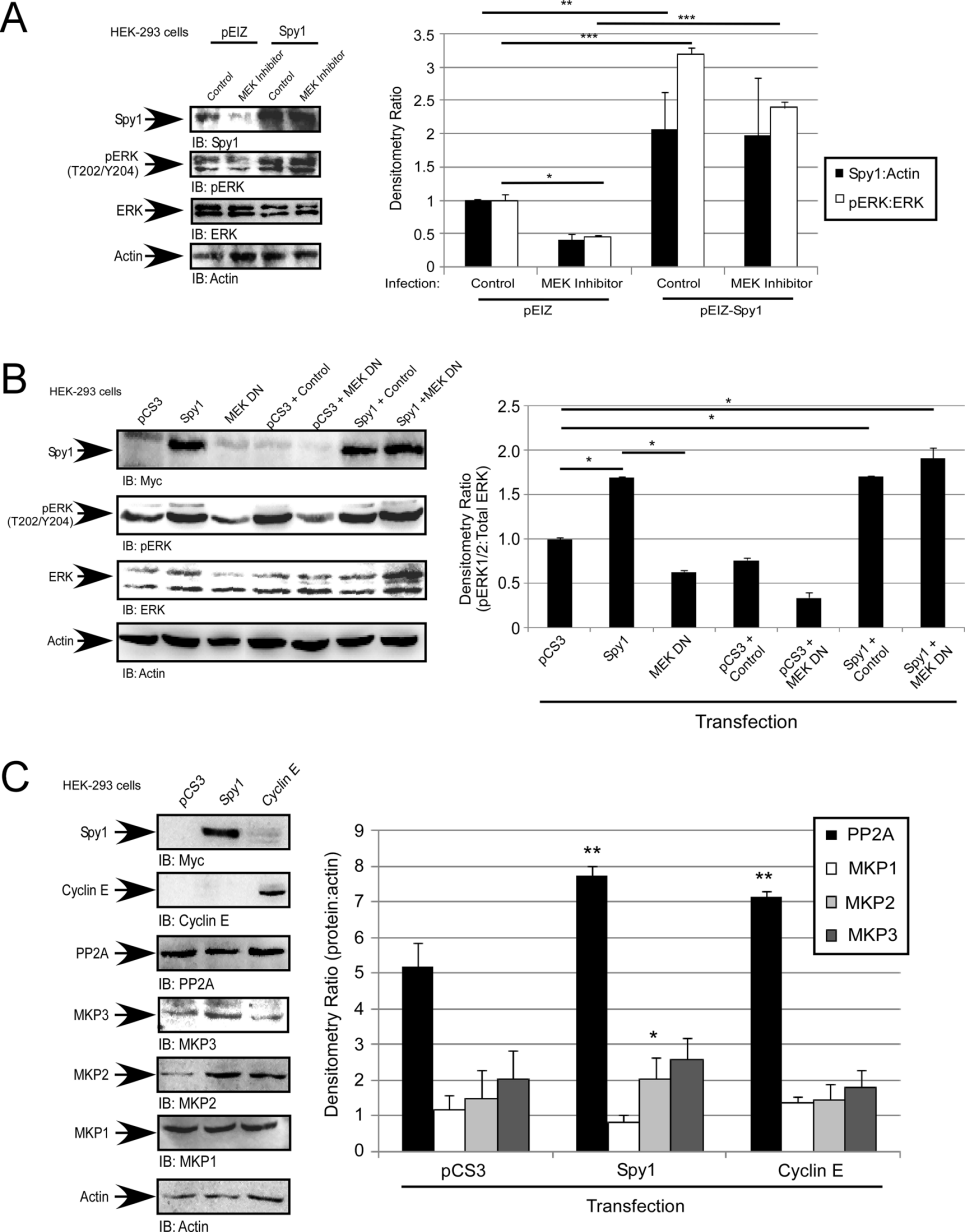
Spy1-mediated ERK phosphorylation is MEK-independent (**A**–**C**) Hek-293 cells were infected (A) or transfected (B and C) with the indicated constructs (along top of each representative blot and X-axis of each graph. (A) Cells were treated with 10 μM U0126 or vehicle control (DMSO). (A–C) Representative blot (left). Densitometry (right) as represented on Y-axis or text box. Error bars reflect SE between at least 3 experiments. Student's *t*-test was performed; *p < 0.05, **p < 0.002, ***p < 0.001.

In the breast, Spy1 levels are elevated by MAPK/ERK and c-Myc signalling to promote proliferation and override differentiation stimuli [13, 16]. In the presence of U0126, a MEK1/2 inhibitor, we see a decrease in endogenous pERK1/2, as well as a significant decrease in overall endogenous Spy1 protein levels, as seen previously [13]. In cells overexpressing Spy1, U0126 does not significantly reduce the ability of Spy1 to activate ERK1/2 (Figure 3A), suggesting that Spy1 activates ERK1/2 independent of MEK. Using a MEK1/2 DN in combination with Spy1 overexpression, pERK continues to be significantly activated, further supporting a MEK-independent mode of activation (Figure 3B). Another level of regulation of ERK1/2 phosphorylation is through the steady state removal of phosphorylation by the relevant phosphatases. Four major phosphatases regulate ERK1/2; PP2A, MAPK Phosphatase (MKP)1, MKP2, and MKP3 [52]. We questioned whether Spy1 expression could alter the expression level of any of these phosphatases. As seen in Figure 3C, neither Spy1 or Cyclin E1 overexpression significantly decreased the protein levels of these phosphatases, Spy1 actually increased PP2A and MKP2 protein levels. It is intriguing to hypothesize that this is a cellular response to retain steady state due to enhanced activation of ERK1/2.

### Spy1 activation of ERK is dependent on Ras and Raf

**Figure 4:**
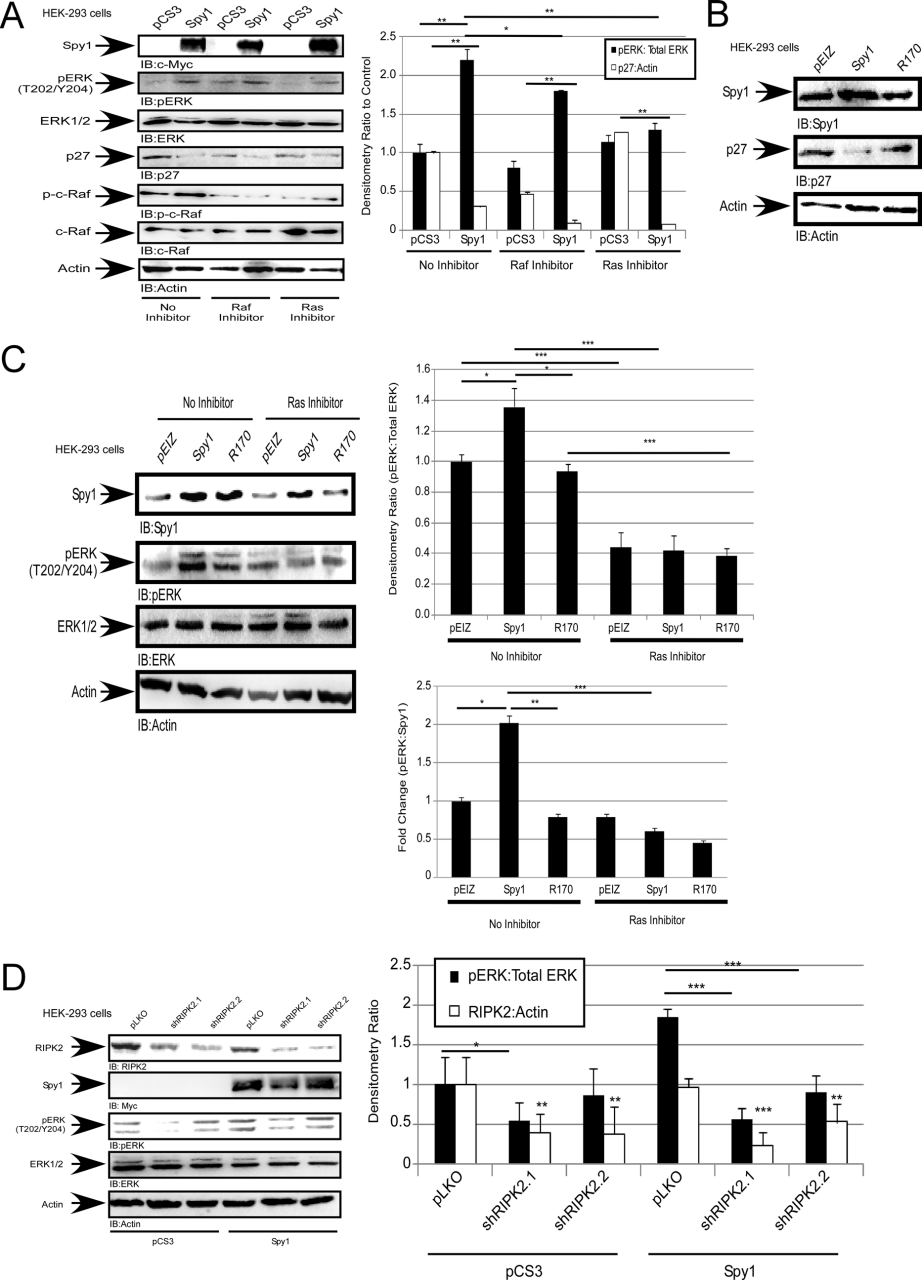
Spy1 activation of ERK1/2 is dependent on Ras and Raf Hek-293 cells were transfected (**A**) or infected (**B**–**D**) with constructs indicated at the top of each blot (left). (A and C) were treated with inhibitors as indicated on the panels. (A, C–D) Densitometry of relative protein levels conducted over all experiments (right). Lower right panel (C) fold change ratio of pERK:Spy1 protein levels. Error bars reflect SE between at least 3 experiments. Student's *t*-test was performed;*p < 0.05,**p < 0.01, ***p < 0.001.

To determine whether alternate upstream activators of ERK1/2 are important in Spy1-mediated effects we tested the consequences of Spy1 overexpression in the presence of Ras and c-Raf inhibitors, Farnesyl Thiosalicyclic Acid and GW5074, respectively. When c-Raf or Ras are inhibited Spy1 is no longer able to activate ERK as compared to the control, indicating that the regulation of pERK by Spy1 requires both Raf and Ras activation (Figure 4A). Moeller et al. (2003) showed Ras activation requires the inhibition of p27. Binding of p27 to Grb2 blocks the interaction of Grb2 with the guanine nucleotide exchange factor, SOS, thereby, inhibiting the formation of the Grb2/SOS complex, which is required to activate Ras [53]. Spy1-CDK2 can bind to and directly inhibit p27 [8, 19]. This work shows that Spy1 inhibits p27 levels in the absence and presence of Raf/Ras inhibitors (Figure 4A, hollow bars), demonstrating that this aspect of Spy1 activity is intact and that ERK-mediated effects reside downstream of p27 degradation. To investigate the importance of the direct Spy1-p27 interaction, a Spy1-p27 binding mutant (R170) was utilized [54]. Spy1-R170 prevents the downregulation of endogenous p27 seen with Spy1-WT as previously published (Figure 4B) [54]. Interestingly, when cells were infected with R170 lentivirus there was no statistical increase in ERK phosphorylation as seen with Spy1-WT (Figure 4C and Supplementary Figure 2). Collectively, these data show the Spy1-CDK complex requires the activation of Ras and depends on the direct interaction with p27 to significantly increase pERK protein levels.

### Spy1 overexpression may function through RIPK2

Receptor-interacting serine-threonine kinase 2 (RIPK2) is phosphorylated and activated through Ras-activated Raf kinase [55]. One established downstream substrate of RIPK2 is ERK1/2 *in vitro* and *in vivo* [55]. To determine whether the effects of Spy1 overexpression on ERK1/2 could be mediated through RIPK2, cells were transfected with Myc-tagged-Spy1 followed by infection with lentivirus packaging either scrambled control shRNA (pLKO) or shRNA targeting two different regions of the RIPK2 mRNA (shRIPK2.1, shRIPK2.2) (Figure 4D). In the presence of RIPK2 knockdown, a decrease in pERK1/2 levels was seen in comparison to Spy1 overexpression alone (Figure 4D). These results demonstrate that Spy1-CDK1/2 can activate ERK1/2 indirectly through the Ras-Raf-RIPK2 pathway.

### Increased levels of Spy1 reduce sensitivity to tamoxifen

**Figure 5:**
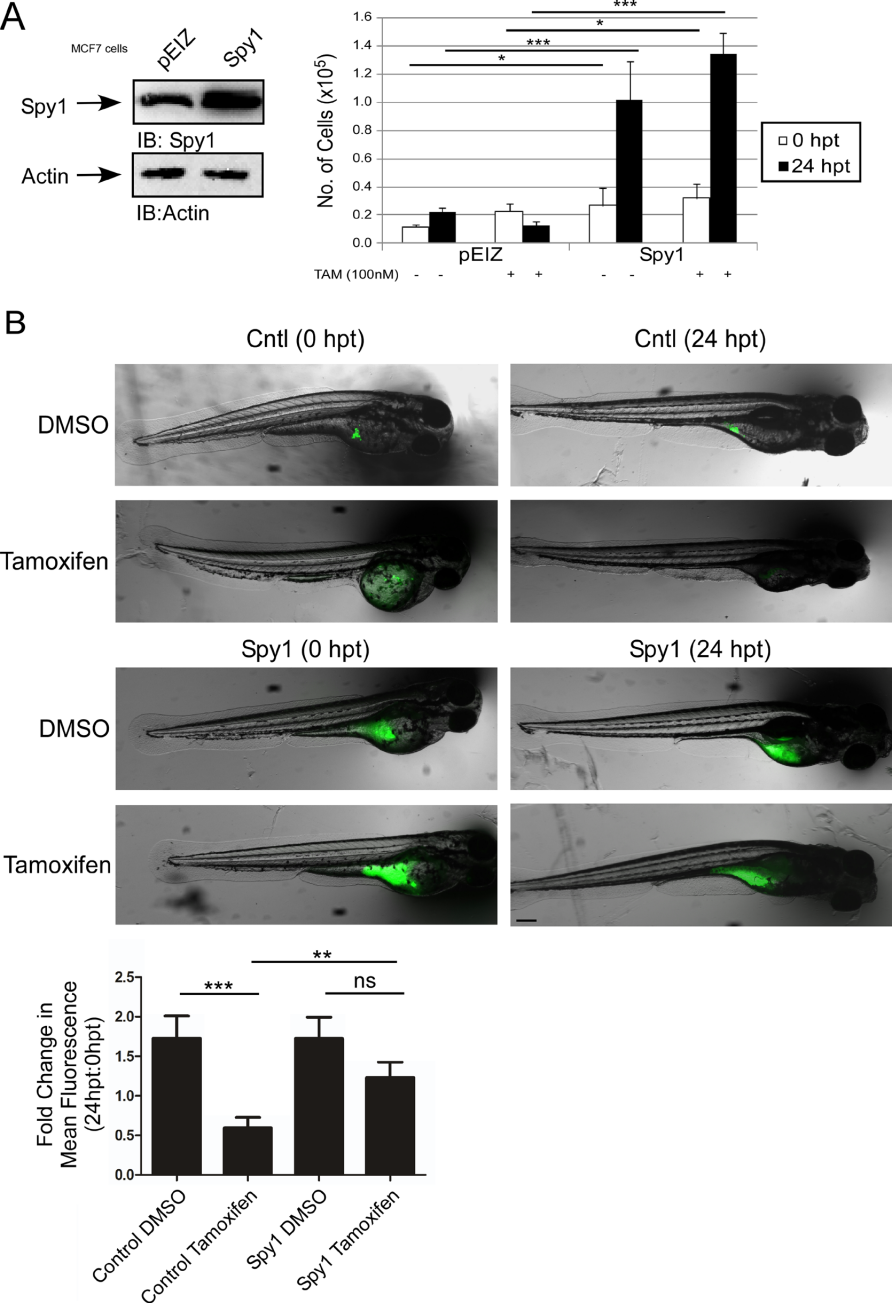
Spy1 levels affect tamoxifen response *in vivo* (**A**) MCF7 cells were infected with indicated constructs (along top of representative blot and X-axis of graph). Trypan blue exclusion assay was performed over indicated time course in the presence or absence of tamoxifen. Error bars reflect SE between triplicate experiments. (**B**) Representative images of injected zebrafish larvae expressing either empty control vector (top panel) or Spy1 overexpression vector (bottom panel) before (0 hpt) and after (24 hpt) treatment with either DMSO or 10 μM tamoxifen. *The same fish is depicted at 0 and 24 hpt for each condition. Scale bar = 200 μm. Graph representing the mean fold change in foci, as quantified by fluorescence as compared to 0 hpt. *n* = 28–46 fish/treatment (excluding mortalities). Student′s *t*-test was performed; ns = not significant, **p < 0.01, ***p < 0.001. Scale bar = 200 μm.

Spy1 is found at elevated levels in aggressive forms of breast cancer [12]. Given the role of ERK signalling in driving tamoxifen resistance [33, 56], we tested the effect of Spy1 levels on the tamoxifen response. ER positive MCF7 cells were infected with pEIZ-Spy1 or empty vector control (pEIZ) (Figure 5A). Cells were treated with 100 nM tamoxifen for 24 hours and subjected to the trypan blue exclusion assay. Spy1 overexpression significantly increased cell number as compared to pEIZ control in untreated cells, as has been previously published [57]. Interestingly, when tamoxifen was added, Spy1 continued to drive cell proliferation, but pEIZ control populations failed to proliferate (Figure 5A), supporting that elevated levels of Spy1 reduced sensitivity to tamoxifen. We implemented a zebrafish xenograft model to elucidate whether Spy1 levels can increase or decrease sensitivity to tamoxifen *in vivo*. The use of zebrafish xenograft models in cancer and drug therapeutics discovery has been recognized and validated as a suitable alternative to mammalian models, with several important advantages [58]. Drug toxicity studies show reliable screening in the zebrafish and that this model is a suitable predictive alternative to mammalian systems [59, 60]. Furthermore, key cell cycle mediators, tumour suppressors, oncogenes, and estrogen-responsive genes and activation of downstream pathways of estrogen signalling are all highly conserved in the zebrafish [61]. Additionally, injection of human breast cells followed by therapeutic drug testing in zebrafish is more efficient than using immunocompromised mice since the adaptive immune system does not develop until 14 days post fertilization (dpf) and, hence, tumour responses can be studied in the presence of residual immune competence [62]. The zebrafish model also allows for high throughput testing of drug combinations in a short time frame, being both cost and time efficient. To validate the model, fluorescently labelled MCF7 or tamoxifen resistant LCC9 cells [63] were injected into embryos at 48 hours post-fertilization (hpf) and treated with tamoxifen for 24 hours (Supplementary Figure 3). Fish were imaged and quantified before and after treatment to determine the changes in number of tumour foci per fish, as quantified by total fluorescence. Tumour foci at 24 hours post-treatment (hpt) demonstrate that while the MCF7 cells respond to treatment, the LCC9 cells are resistant to tamoxifen treatment (Supplementary Figure 3). Sensitive MCF7 cells were then infected with pEIZ control or pEIZ-Spy1 vectors and injected into zebrafish embryos (Figure 5B). Vehicle control (DMSO) or tamoxifen was administered 48 hours post-implantation (hpi) and tumour foci were imaged for each fish at 0 hpt and 24 hpt. The fold change in tumour foci was recorded for each individual embryo to control for variation in injected cell number (quantification lower graph). Tamoxifen had a significant effect on overall cell numbers in control infected cells, which may be a combination of cytostatic and cell death effects. However, when Spy1 was overexpressed there was a significant decrease in sensitivity to tamoxifen treatment *in vivo* (Figure 5B; lower panel).

### Targeting Spy1-directed ERK activation sensitizes cells to tamoxifen

**Figure 6:**
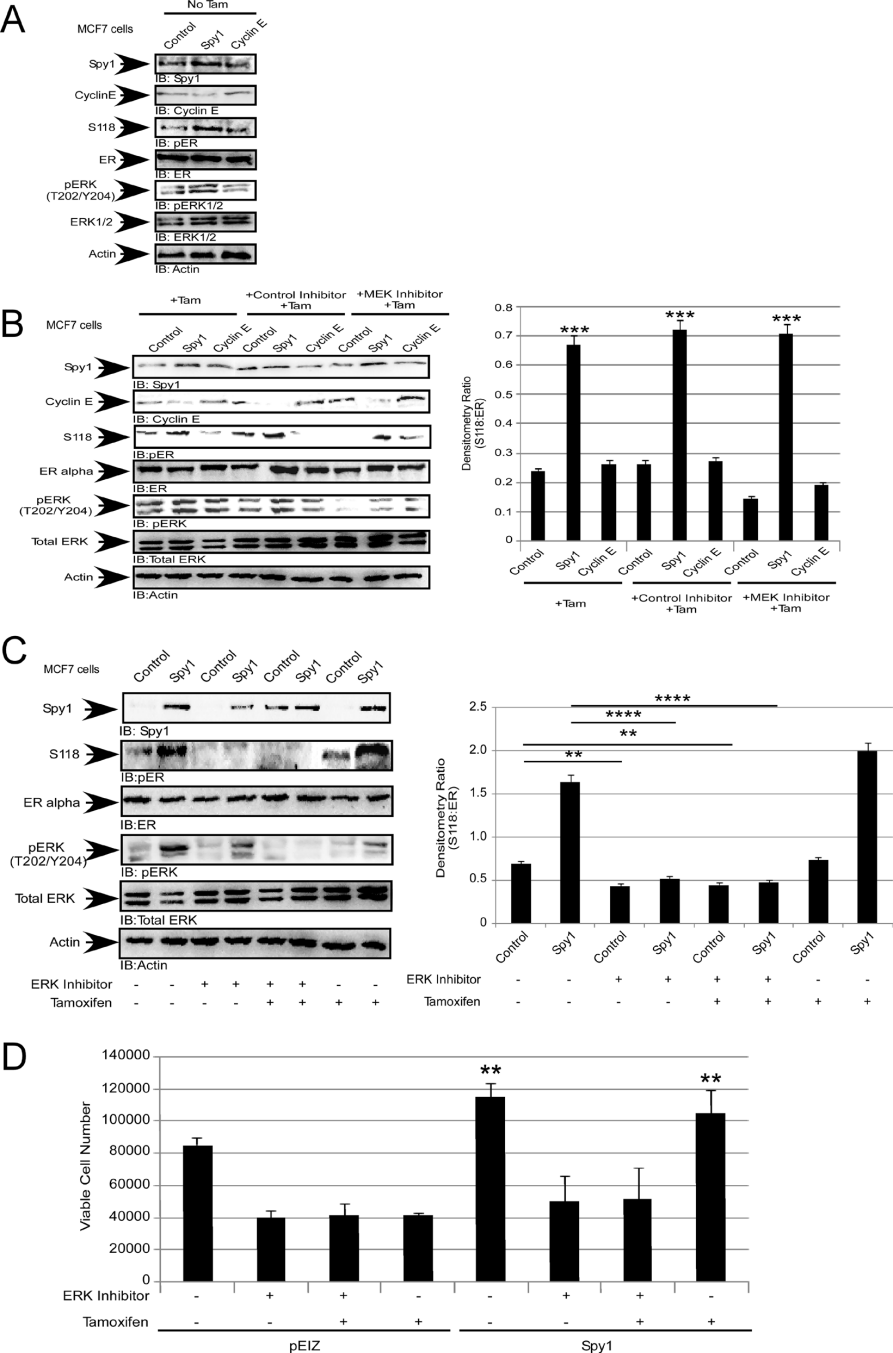
Spy1 levels regulate the response to tamoxifen MCF7 cells were infected with constructs indicated at the top of each representative blot and X-axis of each densitometry graph. Drug treatments are indicated in each panel. Tamoxifen (100 nM), MEK1/2 inhibitor (10 μM), ERK inhibitor (10 μM) and control MEK/ERK inhibitors (10 μM). (**A**) Representative blot (left) of pS118 without tamoxifen treatment. (**B**) Representative blot (left) and quantification of pS118 (right). (**C**) Representative blot (left) and quantification of pS118 (right). (**D**) Viable cell numbers after treatment assayed using trypan blue exclusion. Error bars reflect SE between at least 3 individual experiments. Student's *t*-test was performed; *p < 0.05, **p < 0.01, ***p < 0.001, ****p < 0.0001.

We have demonstrated that Spy1 activates ERK, and subsequent phosphorylation of ERα-S118 in a MEK-independent, but Ras-dependent, fashion (Figures 3, 4). To test whether Spy1 effects on tamoxifen sensitivity were mediated through this pathway, MCF7 cells were treated with tamoxifen in the presence or absence of the MEK inhibitor U0126. When Spy1 levels were elevated, even in the presence of tamoxifen, the levels of pERα-S118 were significantly increased as compared to control and Cyclin E1overexpression and this occurred in a MEK-independent fashion (Figure 6A–6B). We then measured the effect of direct inhibition of ERK1/2 on Spy1-mediated effects on pERα-S118. MCF7 cells overexpressing Spy1 or control were treated with 10 μM ERK1/2 inhibitor (SCH772984), either alone or in combination with 100 nM tamoxifen (Figure 6C). Spy1 significantly increases pERα-S118 in control situations but not in the presence of the ERK inhibitor (with or without Tamoxifen). Cells were then counted for viability after treatment using the trypan blue exclusion assay. We show that the use of the ERK1/2 inhibitor alone significantly reduces the number of viable cells in both control and Spy1 overexpressing cells (Figure 6D). Importantly, Spy1-mediated proliferation was not abrogated by tamoxifen alone but ERK inhibition prevented Spy1-mediated effects on growth (Figure 6D; tamoxifen lanes).

Collectively, these data show that Spy1, an atypical cell cycle protein expressed at elevated levels in many human cancers, alters the post-translational status of the ER and abrogates response to hormone therapy through ERK1/2 activation. Novel therapies focusing on the direct inhibition of ERK1/2 in patient populations harbouring elevated levels of Spy1 may represent a novel therapeutic direction for both treating drug resistant patients and preventing/decreasing the incidence of resistance in ER-positive patients.

## DISCUSSION

In breast cancer estrogen can independently regulate the expression and function of the proto-oncogene c-Myc followed by a rapid activation of the G1 CDK, CDK2, to induce cell cycle progression [25, 64, 65]. Previous reports have perplexed that following estrogen treatment there is little or no change in the levels of Cyclin E, CDK2 or in the formation of cyclin E–CDK2 complexes prior to entry into S phase [66]. This work demonstrates that the atypical cyclin partner for CDK2, Spy1, is transiently upregulated downstream of activated ERα. This reveals a novel pathway for exploration important both to normal development and a host of pathologies. It is interesting to note that a shift in the mobility of Spy1 was seen upon the addition of E2. Whether this is due to a post-translational modification on Spy1 will require further examination. Additionally, we have shown that Spy1 can induce a unique activation of ERK1/2 that may in part dictate ER-mediated proliferation in some ER+ breast cancer cells.

It is important to note that post-translational modification of the ERK1/2 complex is a transient and highly dynamic process [67]. Our data demonstrates that at any specific time breast cancer cells with elevated levels of Spy1 have higher activated ERK1/2 as measured by a general phospho-antibody. Our data further suggests that this is indirect, introducing even more variability in the dynamics of the event. Further investigation into the specific phosphorylation sites on each ERK isoform showed a significant increase in the level of phosphorylation on both sites of ERK2 (T185 and Y187), but only one site of ERK1 (T202). More work would be required to determine if there is any site specificity. It is enticing to consider that perhaps Spy1-CDK mediated signalling could have some direct effects on the post-translational status of the MAPK pathway or direct effectors. Interestingly, Spy1-CDK activation of ERK is unique to Spy1 in that Cyclin E overexpression did not demonstrate a statistically significant change in ERK activation over multiple repetitions. It is notable that we have seen variability in the effects of Cyclin E on ERK activation that do not preclude that Cyclin E may activate ERK under specific experimental conditions.

ERα proliferative signalling in breast cancer cells is dependent upon ligand binding and/or post-translational modification to enable signalling in the absence of ligand. Post-translational modifications to ERα can render the receptor ligand-independent and resistant to anti-estrogen therapies. Phosphorylation on S118 of ERα is one such modification that can lead to tamoxifen resistance [51]. Cellular cycling influences the phosphorylation status of ERα in a manner that is dependent upon mitogen stimulation [25, 68]. In particular, phosphorylation on S118 is regulated by activated ERK1/2 [33, 34, 51]. This work demonstrates that Spy1 levels correlate with an increase in phosphorylation of S118 on ERα in a manner dependent on ERK1/2 activity, thereby providing a direct link between ER modification and the cell cycle machinery.

**Figure 7:**
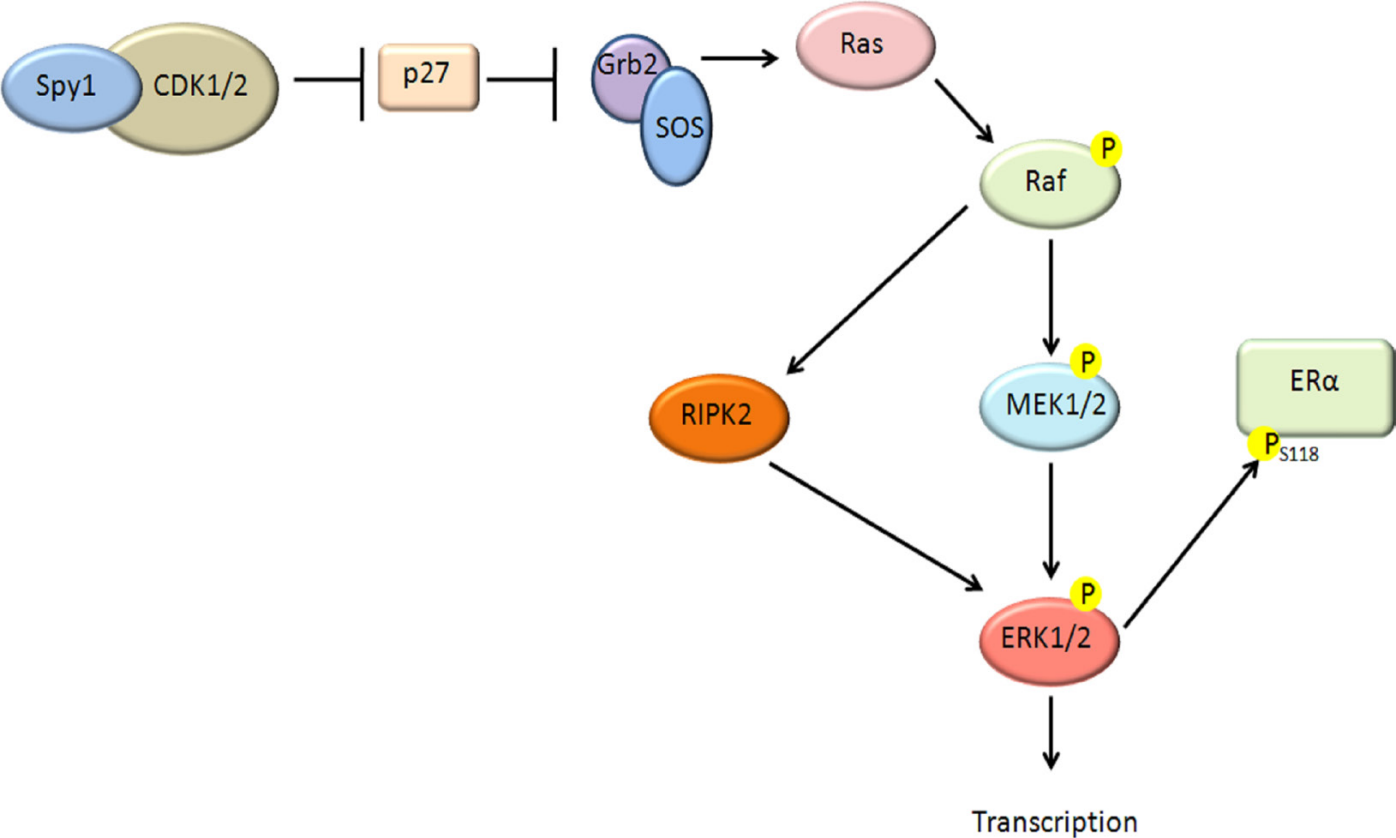
Schematic diagram of proposed pathway Our data supports that elevated levels of Spy1 can act upstream to activate ERK1/2 through a MEK-independent pathway. Our data supports that this depends on a direct interaction with p27, as well as Ras and Raf activation. A novel pathway has been described demonstrating a Raf downstream kinase, RIPK2 [55] capable of activating ERK1/2 in a MEK-independent fashion. Our data shows a dependence of Spy1-mediated effects on RIPK2. Spy1-mediated ERK1/2 activation can phosphorylate the ER on S118 and alter sensitivity to tamoxifen.

Lenormand et al. (1999) showed that Spy1 could activate MAPK in *Xenopus* oocytes [16]. Unlike the Lenormand data, however, Spy1 activation of ERK1/2 in the human breast cell line, MCF7, appears to be independent of MEK, and at least in part dependent on the direct interaction between Spy1 and the cell cycle inhibitor p27. Our work is not the first to show a MEK-independent activation of ERK1/2, Aksamitiene et al. (2010), have demonstrated that ERK1/2 can be activated in a MEK-independent, but PI3K/Akt-sensitive fashion, in the ER-positive breast cancer cell line T47D [69]. Our data supports that upstream pathways driven by Spy1 may converge on an activation of the novel ERK activator RIPK2 (See Figure 7).

Mutations within Ras and Raf, or hyperactivation of MEK1/2 has spurred the production of pharmacological inhibitors targeting mediators upstream of ERK1/2; however, resistance and relapse occurs within 6 to 7 months of treatment [45]. Our findings may suggest that in some cancers with an elevated Spy1 overexpression small molecule inhibitors for MEK1/2 may be ineffective. Indeed, we have shown elevated levels of Spy1 alone can override MEK1/2 inhibitors and increase the phosphorylation status of ER on S118. Since ERK1/2 activation significantly promotes uncontrolled cell growth, survival, and invasion, determining a way to suspend ERK1/2 activation by Spy1 could have clinical implications for tamoxifen resistance and breast cancer therapies in at least subsets of patients. A significant decrease in cell viability and a concomitant decrease in phosphorylation of ERα-S118 in the presence of elevated Spy1 was seen only when ERK1/2 was directly inhibited. The discovery of a new inhibitor specifically targeting the ATP-binding site of ERK1/2 has shown promising results with respect to solid tumours [45, 46]. Indeed, our data herein shows that inhibition of ERK1/2 results in an abrogation of S118-ERα phosphorylation and a significant restoration of response to tamoxifen treatment in ER+ cells with elevated Spy1 levels. Hence, therapies focusing on the direct inhibition of ERK1/2 in ER+ patient populations harbouring elevated levels of Spy1 may represent an effective therapeutic direction for preventing/decreasing resistance to hormone therapy.

## MATERIALS AND METHODS

### Cell culture

Human embryonic kidney (HEK)-293 and MCF7 cells were purchased from ATCC and were subcultured in DMEM media supplemented with 10% FBS and 30,000 units penicillin/30,000 μg streptomycin solution. Cells were maintained under normoxic conditions (5% CO_2_) at 37°C. LCC9 cells (Lombardi Comprehensive Cancer Center, Georgetown University) were routinely subcultured in DMEM phenol red free media supplemented with 1 mM L-glutamine, 30,000 units penicillin/30,000 μgstreptomycin,and 10% charcoal treated FBS. Cells were maintained under normoxic conditions (5% CO_2_) at 37°C.

### Plasmids

Creation of the Myc-Spy1-pCS3 was described previously [57]. Plasmids for Rc-CMV-Cyclin E (#8963), pEGFP-C1-ERα (#28230), HA-CDK1-DN (#1889), DN9 (Mek1 dominant negative; #21209), and pLKO-scrambled control (#8453) were purchased from Addgene. pLKO-shSpy1 and pLKO-shCyclin E were cloned to express a short hairpin previously described to knockdown Spy1 and Cyclin E respectively and are previously described [7]. Control pLKOcontains a scrambled sequence previously described [7]. The CDK mutants D90 [17] and R170 vectors [54] have been previously described. pEIZ vector was generously donated from Dr. B. Welm (Univ. of Utah). The creation of pEIZ-Spy1 was completed by inserting Spy1 oligo into the *Eco*RI and *Xba*I sites of pEIZ.

### Immunoblotting (IB)

Whole cell lysates were prepared as described previously [12] and aliquots of lysates containing 100 μg protein were subjected to electrophoresis on denaturing 10% SDS polyacrylamide gels and transferred to PVDF-Plus 0.45 micron transfer membrane (Osmonics Inc.) for 2 hours at 30 volts using a wet transfer method. Chemiluminescent Peroxidase Substrate was used for visualization following manufacturer's instruction (Pierce). Chemiluminescence was quantified on an AlphaInnotech HD2 (Fisher) using AlphaEase FC software.

### Antibodies

Actin was purchased from Chemicon-Millipore (MAB150 1R). Spy1 was purchased from ThermoScientific (PA5-29417). c-Myc (9E10, C3956)and secondary rabbit and mouse antibodies were purchased from Sigma. Phospho-ERα-S118 (ab32396), Cyclin E1 (ab33911), phospho-Raf1 (ab135559), p27 (ab7961), anti-ERK1 (phospho T202) + ERK2 (phospho T185) (ab201015), and anti-ERK1 (phospho Y204) + ERK2 (phospho Y187) (ab47339) were purchased from Abcam. ERα (sc-543), RIPK2 (sc-8610), ERK1/2 (sc-154), MKP1 (sc-271684), MKP2 (sc-1200), MKP3 (sc-8598), PP2A (sc-6110), and Raf1 (sc-7267) were purchased from Santa Cruz Biotechnology. Phospho-ERK 1/2 (Thr 202/Tyr 204)was purchased from Cell Signaling (4370).

### Transfection/infection

#### Transfection

Cells were transfected using polyethylenimine (PEI) branched reagent (Sigma, 408727). In brief, 10 μg of DNA was mixed with 3 μl of 10 mg/ml PEI for 10 minutes then added to a 10 cm tissue culture plate. Transfection media was changed after 24 hours.

### Infection

8000 cells were seeded in fully supplemented growth media in 96-well plates for 2 hours. Cells were starved by removing serum and penicillin/streptomycin from the media, followed by the use of 1 mg/ml polybrene (Santa Cruz, sc-134220) and MOI 3 of the specific vector used. Infected media was changed to fully supplemented media 24 hours after infection. For knockdown, cells were incubated with 1mg/ml puromycin (Sigma, P8833) 48 hours after infection for 72 hours to allow for puromycin selection. Media is thereafter changed every 48hours with puromycin included.

### Inhibition treatments

HEK-293 cells were seeded equally in 10 cm dishes at a density of 5 × 10^5^ cells. Upon 80% confluency, HEK-293 cells were incubated with either 10 μM SB202474 (control; EMD Millipore, 559387) or 10 μM U0126 (MEK 1/2; EMD Millipore, 662005) inhibitors for 1 hour. For Raf inhibition, 5 μM GW5074 (Sigma, G6416) was added to the cells for 24 hours. For Ras inhibition, 20 μM Farnesyl Thiosalicyclic Acid (Santa Cruz, sc-205322) was added to the cells for 24 hours. For ERK1/2 inhibition, 10 μM SCH772984 (ApexBio, A3805) was added to the cells for 1 hour prior to treatment with tamoxifen (Sigma, H7904) for 24 hours. Following drug treatment, cells were either analyzed using trypan blue analysis or pelleted, lysed and analyzed using 10% SDS-PAGE.

### Estradiol treatments

MCF7 were seeded equally in 10 cm dishes at a density of 5 × 10^5^ cells. Upon 70% confluency, the cells were treated with phenol red-free RPMI media, supplemented with 10% charcoal treated FBS and 30,000 units penicillin/streptomycin. After 48 hours, cells were incubated with either dimethyl sulfoxide (DMSO) or 50 ng/ml E2 (Sigma, E8875) for specified time points, followed by cell harvesting for protein extraction and IB.

### Animal care and handling

Wildtype Zebrafish (*Daniorerio)* were handled in compliance with local animal care regulations and standard protocols of Canada and following the University of Windsor animal care protocol #12–14. Adult fish were kept at 28.5°C and bred according to available protocols [56].

### Implantation and treatment

Eggs were collected after fertilization and kept in E3 embryo media (5 mM NaCl, 0.17 mMKCl, 0.33 mM CaCl_2_, 0.33mM MgSO_4_, 10^−5^% Methylene Blue) at 32°C in an incubator until ready to inject. Before injection 200,000 cells were reconstituted in 200 μL of serum-free media and labelled with 1 μL of DiO (green) (Vybrant, Invitrogen) at 37°C for 20 minutes. Cells were washed with 200 μL of serum free media twice and resuspended in 20 μL of serum free media, kept at 37°C for 20 minutes, and placed on ice until injection. 48 hours post-fertilization (hpf) the embryos were dechorionated with fine tip forceps and anesthetised with 0.168 mg/ml of Tricaine (Sigma, MS222). 50–100 labelled cells/ 9 nL were loaded into glass capillary needles and injected into the yolk sac of each embryo using a Nanoject II (Fisher Scientific). After injection, embryos were placed in E3 embryo media and 2 hours post-implantation (hpi) were examined using a Leica fluorescence stereoscope to exclude any embryo with cells outside of the implantation area. Mortalities due to injection will also be evident at this time point for exclusion (~5–15%). 24 hpi (0 hours post-treatment (hpt)) the embryos were anesthetized, imaged and placed in a 96-well plate; one embryo per well. At 48 hpi the embryos were treated with either DMSO or 10 μM tamoxifen. The embryos were imaged at 0 hpt and 24 hpt and the fold change in tumour foci were quantified by total fluorescence.

### Image analysis

The fish were imaged before and after treatment with Tamoxifen and DMSO. The image for each embryo was imported into ImageJ, the image was converted to a 32-bit greyscale, and the threshold was adjusted to eliminate background pixels. Total area of fluorescence was measured as the tumour area and the ‘particle analysis’ function was used to calculate number of tumour foci. Using automated software we aligned the site of injection over multiple fish to determine overall changes in the distance that tumour foci are detected from injection site. All measured results were copied into Excel files and fold change in tumour area and tumour foci calculated from 24 to 72 hpi.

## SUPPLEMENTARY MATERIALS FIGURES AND TABLES


