# Supplementary material for: The cyclin-like protein, SPY1, regulates the ERα and ERK1/2 pathways promoting tamoxifen resistance
Source: Oncotarget. 2017 Feb 21;8(14):23337–52. doi: 10.18632/oncotarget.15578 (PMC5410308; doi:10.18632/oncotarget.15578)
Supplement: Supplementary file 1 [file oncotarget-08-23337-s001.pdf]

The cyclin-like protein, SPY1, regulates the ERα and ERK1/2 pathways promoting tamoxifen resistance

Supplementary Materials

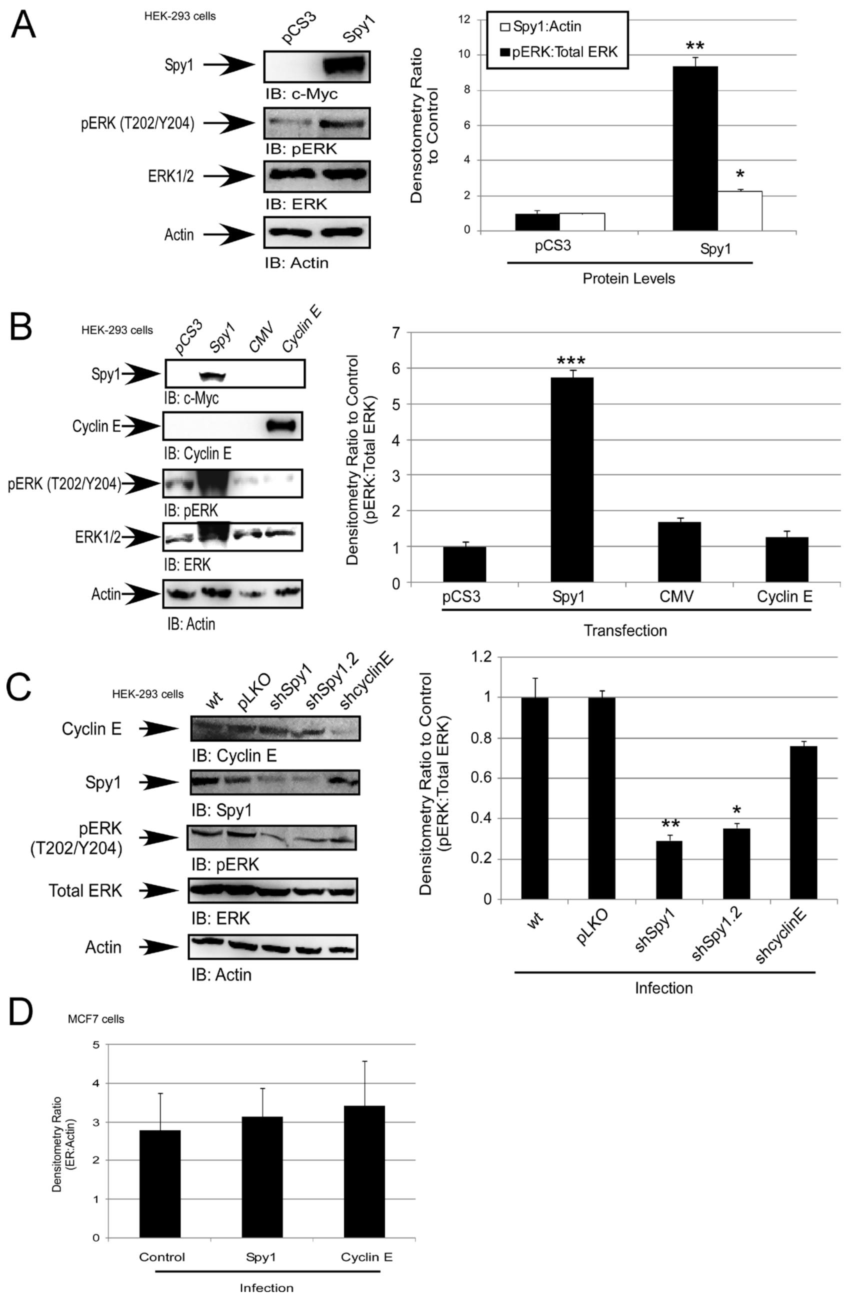

Supplementary Figure 1: Spy1 overexpression enhances ERK1/2 phosphorylation. (**A–D**) Cells transfected or infected with the indicated constructs, followed by SDS-PAGE and IB. Representative blots are depicted on the left. Densitometry was conducted and averaged over triplicate experiments (right). All protein levels are normalized to actin.Error bars reflect SE between multiple experiments. Student's *t*-test was performed; $*p < 0.05$, $**p < 0.01$, $***p < 0.001$.

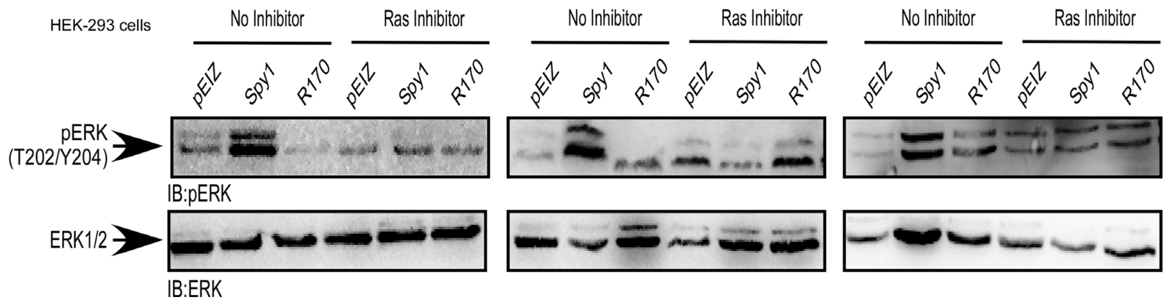

Supplementary Figure 2: Spy1 activation is dependent on Raf and Ras. Replicate representative blots of Hek-293 cells infected with control, Spy1 overexpression, or Spy1-p27 binding mutant in the presence or absence of the Ras Inhibitor.

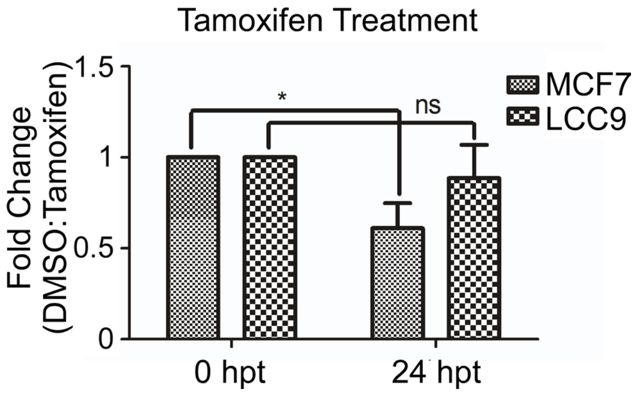

Supplementary Figure 3: *In vivo* response to tamoxifen treatment in tamoxifen-sensitive and -resistant cell lines. Zebrafish were injected with either the same number of MCF7 or LCC9 cells (*n* = 5) for each line. After 24 hr fish were treated with 10 µM tamoxifen or control. The area of tumour foci per fish were counted and expressed as the fold change in comparison to 0 hpt.*$p < 0.05$.hpt = hour post-treatment.
